# Modulation of eDNA Release and Degradation Affects *Staphylococcus aureus* Biofilm Maturation

**DOI:** 10.1371/journal.pone.0005822

**Published:** 2009-06-09

**Authors:** Ethan E. Mann, Kelly C. Rice, Blaise R. Boles, Jennifer L. Endres, Dev Ranjit, Lakshmi Chandramohan, Laura H. Tsang, Mark S. Smeltzer, Alexander R. Horswill, Kenneth W. Bayles

**Affiliations:** 1 Department of Pathology & Microbiology, University of Nebraska Medical Center, Omaha, Nebraska, United States of America; 2 Department of Microbiology and Cell Science, University of Florida, Gainesville, Florida, United States of America; 3 Department of Microbiology, University of Iowa, Iowa City, Iowa, United States of America; 4 Department of Microbiology & Immunology, University of Arkansas for Medical Sciences, Little Rock, Arkansas, United States of America; Columbia University, United States of America

## Abstract

Recent studies have demonstrated a role for *Staphylococcus aureus cidA*-mediated cell lysis and genomic DNA release in biofilm adherence. The current study extends these findings by examining both temporal and additional genetic factors involved in the control of genomic DNA release and degradation during biofilm maturation. Cell lysis and DNA release were found to be critical for biofilm attachment during the initial stages of development and the released DNA (eDNA) remained an important matrix component during biofilm maturation. This study also revealed that an *lrgAB* mutant exhibits increased biofilm adherence and matrix-associated eDNA consistent with its proposed role as an inhibitor of *cidA*-mediated lysis. In flow-cell assays, both *cid* and *lrg* mutations had dramatic effects on biofilm maturation and tower formation. Finally, staphylococcal thermonuclease was shown to be involved in biofilm development as a *nuc* mutant formed a thicker biofilm containing increased levels of matrix-associated eDNA. Together, these findings suggest a model in which the opposing activities of the *cid* and *lrg* gene products control cell lysis and genomic DNA release during biofilm development, while staphylococcal thermonuclease functions to degrade the eDNA, possibly as a means to promote biofilm dispersal.

## Introduction

Bacterial biofilm is defined as an ordered assembly of bacterial cells contained within a polymeric matrix [1], [2]. Not only does the matrix provide a structural and protective physical barrier against harsh environmental conditions such as the host immune response and desiccation, it also provides a physiological niche in which the cells become more resistant to the killing effects of antibacterial agents. Recent studies demonstrate that the biofilm matrix can be comprised of a variety of important structural components including polysaccharides, proteins, and DNA [3]–[9]. The importance of extracellular genomic DNA (eDNA) as a structural component of biofilm was first demonstrated in *Pseudomonas aeruginosa* [10] but has subsequently been demonstrated in a variety of bacterial species using experiments in which eDNA is removed from the biofilm or that utilize lysis defective mutants unable to release normal amounts of genomic DNA into the matrix [6], [7], [11], [Rice, 2007 #22] [12]–[15]. Recent data also suggest that the presence of DNA within the matrix may contribute to the recalcitrance of biofilms to antibiotics by inducing expression of antibiotic resistance genes [16].

The molecular mechanisms facilitating release of eDNA have been studied in *P. aeruginosa* [17], *S. aureus* [7], and *Enterococcus faecalis* [12]. The results of a study by Webb et al. [17] suggest that bacteriophage-mediated cell death and lysis of *P. aeruginosa* promotes microcolony development and dispersal. In experiments with *Enterococcus faecalis*, Thomas et al. [12] demonstrated the effects of two secreted proteases, GelE and SprE, on biofilm development. The GelE protease is the effector of lysis while the SrpE protease was described as an immunity factor that opposes the effects of GelE. It was proposed that a balance between these two proteases affects cell wall hydrolysis and lysis of a subpopulation of cells resulting in release of genomic DNA and enhanced biofilm formation [12]. Although the specific mechanisms controlling cell death and lysis are likely to vary among species, these studies demonstrate that the control of these processes has a significant impact on biofilm development.

In *S. aureus*, cell death and lysis have been shown to be controlled by the *cid* and *lrg* operons, which have opposing effects on murein hydrolase activity and antibiotic tolerance during planktonic growth [18], [19]. The products of the *cidA* and *lrgA* genes are proposed to function as holins and antiholins, respectively, regulating cell lysis in a manner thought to be analogous to that observed during bacteriophage-mediated cell lysis [18], [20], [21]. Expression of the *cid* and *lrg* operons is regulated by the LytSR [22] and CidR [23] regulators which function in response to changes in membrane potential [24] and glucose metabolism-mediated acetic acid accumulation in the culture medium [25], respectively. Recently, the *cidA* gene was shown to promote cell lysis and the release of DNA during the development of a biofilm [7]. The importance of this eDNA in biofilm formation was demonstrated by the observation that biofilm adherence could be reduced by treatment with exogenously added DNase I. These studies suggest that the biological function of the Cid/Lrg system involves the coordinated regulatory control of cell lysis during biofilm development.

In the study presented here, we examined additional elements affecting eDNA levels within a developing *S. aureus* biofilm. Consistent with our previous findings, the *S. aureus cidA* gene displayed a positive role in cell lysis during biofilm development [7] while the *lrg* operon, as an inhibitor of lysis [19], exhibited a negative role. Interestingly, mutations in both of these operons led to aberrant biofilm maturation, indicating that balanced expression of these genes is important in biofilm development. Finally, staphylococcal thermonuclease was also shown to be important in biofilm development, suggesting that the eDNA produced as a result of lysis is countered by nuclease-mediated degradation.

## Materials and Methods

### Bacterial strains and growth conditions

The *Staphylococcus aureus* strains used in this study were derived from the previously characterized osteomyelytis isolate, UAMS-1 [26], and are listed along with plasmids used in Table 1. All the experiments were initiated using fresh overnight cultures grown at 37°C in tryptic soy broth (TSB) (EMD Biosciences, Gibbstown, NJ) using a 10∶1 flask to volume ratio.

**Table 1:** Strains and plasmids used in this study.

Strain or plasmid	Description	Reference
*Escherichia coli*		
DH5α	Host strain for construction of recombinant plasmids	[49]
*Staphylococcus aureus*		
RN4220	Highly transformable strain; restriction-deficient	[50]
UAMS-1	Clinical isolate	[26]
KB1050	UAMS-1 *cidA*:Em	[25]
KB1045	UAMS-1 *lrgAB*::Em	This work
KB1046	UAMS-1 *lrgAB*::Em (pDR45)	This work
UAMS-1471	UAMS-1 Δ*nuc*	[41]
UAMS-1552	UAMS-1471 (pLI50::*nuc*)	[41]
USA300 LAC	USA300-type isolate	[51], [52]
**Plasmids**		
pCR2.1	*E. coli* PCR cloning vector	Invitrogen
pCN50	Shuttle vector conferring Cm^R^	[53]
pCN8298	Low-copy shuttle vector	[53]
pJE04	pCN8298, Cm^R^ replacing Em^R^	This work
pDR45	pJE04::*lrgAB*	This work
pAH9	*sarA* promoter P_1_-RFP, Amp^R^/Erm^R^	[3]

### DNA manipulations

An *lrgAB* mutation was generated in the UAMS-1 background using a deletion plasmid previously used to make the *lrgAB* mutant in strain RN6390 [19]. This plasmid was transformed into *S. aureus* strain RN4220 by electroporation, spread onto tryptic soy agar (TSA) plates containing Erm and incubated at 30°C overnight. The plasmid was then transferred into UAMS-1 by phage-mediated transduction [27]. Transductants were grown at the non-permissive temperature (43°C) in the presence of tetracycline to select for cells in which the plasmid had integrated into the chromosome via homologous recombination. To promote a second recombination event, a single colony was inoculated into antibiotic-free TSB and grown at 30°C for five days after performing 1∶1000 dilutions into fresh antibiotic-free media each day. After the fifth day the culture was diluted and plated on TSA medium to yield isolated colonies. The colonies were then screened for Erm^R^ and Tc^S^. Verification that the *lrgA* and *lrgB* genes had been deleted was carried out by PCR amplification and Southern blot analyses. The confirmed mutant strain was designated KB1045.

Complementation of the *lrgAB* mutation was achieved using a plasmid expressing the *lrgAB* operon using its native promoter. The *S. aureus* sequence for strain MRSA252, which has been shown to be most closely related to UAMS-1 [28], was used to design an oligonucleotide primer (lrgA-pro-BamHI-F; 5′-CGCGGATCCGAATCGTTATGAAAAACGATTGAATCC-3′) that annealed to the sequence starting 308 bp upstream of the *lrgA* locus while inserting a BamHI restriction endonuclease cleavage site at the 5′ end of the fragment. Another primer (lrgB-KpnI-R; 5′-GCGGGTACCTTAGAAGAATATTGCTACAAAGACAGGC-3′) was designed to anneal near the end of the *lrgB* gene and incorporate a KpnI restriction site at the 3′ end of the amplified DNA fragment. After PCR amplification, the *lrgAB*-containing DNA fragment was sub-cloned using the TA cloning kit (Invitrogen, Carlsbad, CA) and pCR2.1 into *E. coli* strain DH5α and subsequently sequenced to ensure that no mutations had been introduced during PCR-amplification. Next, the *lrgAB* insert was excised by digestion with BamHI and KpnI, and inserted into the low-copy vector, pJE04 (Table 1). This resulting complementation plasmid, designated pDR45, was transformed into *S. aureus* RN4220 by electroporation and then into KB1045 generating strain KB1046 (Table 1).

### Static biofilm assays

To determine the sensitivity of biofilms to DNase I and polyanethole sulfonate (PAS), static biofilm assays were performed as described previously [7]. Costar 3596 (Corning Life Sciences, Acton, MA) plates were pre-coated for 24 h with 200 µl of 20% (vol/vol) human plasma (Sigma, St. Louis, MO) in bicarbonate buffer. Wells were inoculated with 200 µl of overnight *S. aureus* cultures diluted in tryptic soy broth (TSB) supplemented with 3.0% (wt/vol) NaCl and 0.5% (wt/vol) glucose (TSBsg) to an OD_600_ of 0.05. Where indicated, 28 U of DNase I per well or 500 µg/ml PAS per well was added. Static biofilms comparing the phenotypes of the UAMS-1, KB1050, KB1045, and KB1046 strains were performed similarly except that 16% plasma was used to coat all the wells, and the media used was supplemented with 0.5% glucose (TSBg). Static biofilms were grown for 24 h and adherence was analyzed by washing twice with 200 µl phosphate buffered saline (PBS), fixing with 100 µl ethanol for 1 min, and staining with 100 µl crystal violet for 1 min as previously described. Biofilm quantification was performed by measuring the amount of crystal violet retained in the wells (at A_595_) with a Victor^3^ Multilabel Counter (Perkin Elmer, Waltham, MA). Isolation of eDNA from static biofilms was performed as described previously [7]. After 24 h, the plates were chilled at 4°C for 15 min, and 1.0 µl of 0.5 M EDTA was added to each well. The supernatants were discarded, and the unwashed biofilms were harvested by resuspension in 50 mM TES buffer (Tris_HCl; pH 8.0/10 mM ETDA/500 mM NaCl) and transferred into pre-chilled tubes. After centrifugation for 5 min at 4°C, 100 µl of each supernatant was transferred to a tube containing 300 µl of TE buffer (10 mM Tris_HCl; pH 8.0/1.0 mM EDTA), and extracted once with an equal volume of phenol/chloroform/isoamyl alcohol (25∶24∶1) and once with chloroform/isoamyl alcohol (24∶1). The aqueous phase of each sample was then mixed with 1/10 volume of 3.0 M Na-acetate (pH 5.2) and three volumes of ice-cold 100% (vol/vol) ethanol and stored at 20°C. The next day, the ethanol-precipitated DNA was collected by centrifugation for 20 min at 4°C and 18,000×*g*, washed with ice-cold 70% (vol/vol) ethanol, air-dried, and dissolved in 20 µl of TE buffer. Q-PCRs were performed on 1∶10 dilutions of each sample with the LightCycler DNA Master SYBR Green I (Roche) using four primer sets [7]. Both the static biofilm assay and eDNA concentration levels were statistically analyzed based on the repeated measures ANOVA method adjusting for gene effects.

### Flow-cell biofilm assays

Flow-cell biofilm assays were performed using an FC271 flow-cell apparatus (Biosurfaces Technology Inc, Bozeman, MT) containing a 2 mm thick polycarbonate coupon and assembled according to manufacturer's instructions. Biofilm formation was initiated by inoculating with overnight cultures that had been adjusted to an OD_600_ of 2.0 and then diluted 1∶1,000 in TSB. A total of 3.0 ml of the diluted culture was injected into the chamber and allowed to incubate statically for 2 hours. After this incubation period, 5% TSB (vol/vol) supplemented with 0.125% glucose was constantly perfused over the biofilm in a once-through system using a Rainin RP-1 Peristaltic Pump (Rainin Instrument LLC, Woburn, MA). Media was pumped at a rate of 0.25 ml/min. For high-inoculum biofilms, the growth conditions were similar except that the media was adjusted to 2% TSB supplemented with 0.2% glucose, and a 1∶100 dilution of overnight culture was used to inoculate the flow-cell chamber.

After three days of biofilm development, macroscopic images were taken using a Canon EOS camera with a macroscopic lens (18–55 mm) (Canon Inc, New York, NY). For confocal laser scanning microscopy (CLSM) analysis, the media flow was stopped and fluorescent dyes were injected into the flow-cell chamber. Syto-9 (1.3 µM final concentration) was applied to identify viable cells in the biofilm, propidium iodide (PI; 4.0 µM final concentration) was added to stain dead cells, and Toto-3 (2.0 µM final concentration) was used to stain both dead cells and eDNA. The media tubing was then clamped and the biofilm was imaged using a Zeiss 510 Meta CLSM with an Achroplan 40×0.8 n.a. water dipping objective. The Syto-9 and PI fluorophores were exited with an argon laser at 488 nm, and the emission band-pass filters used for Syto-9 and PI were 515±15 nm and 630±15 nm, respectively. Excitation of Toto-3 was achieved using a HeNe 633 nm laser and emissions were collected using a 680±30 nm filter. Examination of high-inoculum biofilms was performed using strains each containing pAH9 which confers RFP fluorescence (Table 1) using a Radiance 2100 system (Biorad) with a Nikon Eclipse E600 microscope. Z-stacks were collected at 1.0 µm intervals and the images were compiled to generate three-dimensional renderings. All confocal parameters were set using wild-type biofilm and were used as standard settings for comparison to the biofilms produced by the mutant and complementation strains. Regions of interest within the biofilms were selected from similar areas within each flow-cell chamber and each confocal experiment was repeated a minimum of four times. CLSM z-stack processing was performed using both the Zeiss ZEN LE software package (Carl Zeiss, Jena, Germany) and the Volocity software (Improvision, Lexington, MA). Measurements of the biofilms produced were performed using the COMSTAT software package [29] calculating the biomass, thickness, and roughness coefficients of the biofilm data accumulated for at least three separate image z-stacks.

Sensitivity of mature, low-inoculum biofilms to DNase I was assessed by growing the biofilms in an FC271 flow-cell apparatus as described above. After one or three days of biofilm development, a mixture of RNase-free DNase I (Qiagen, Valencia, CA; final concentration of 0.5 knitzU•ml^−1^) and 20 µM CaCl_2_ was added to the growth medium and biofilm growth was continued for an additional six hours. High-inoculum biofilms were grown for two days before the addition of RNase-free DNase I and 20 µM CaCl_2_ for a total of three days. All macroscopic images were taken using a Canon EOS camera with a macroscopic lens as described above.

## Results

### Early lysis is important for biofilm adherence

**Figure 1:**
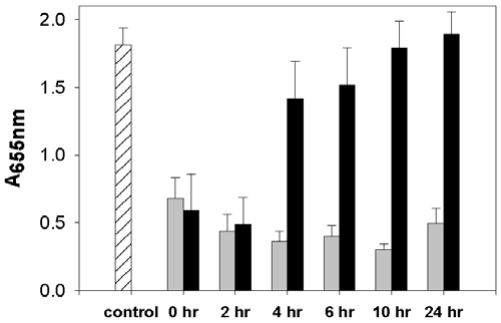
Extracellular DNA-mediated attachment of static biofilm. *S. aureus* UAMS-1 static biofilms were treated with either DNase I (grey bars) or PAS (black bars) at the time of inoculation (t = 0), and at 2, 4, 6, 10, and 24 hours post-inoculation. All biofilms were grown at 37°C for a total of 24 hours. 24-hour biofilms were allowed to grow for several hours after PAS or DNase I addition, to allow full penetration and activity of the compound on the biofilm. The biofilms were washed, stained with crystal violet, and retained biomass was quantified by measuring the absorbance of each well at an absorbance of 655 nm. Mean values from three independent experiments are shown and error bars represent the SEM.

It has been established that eDNA released as a result of cell lysis aids in the formation of an adherent staphylococcal biofilm [6], [7], [14], [30]. To probe the temporal requirements for lysis during the initial stages of biofilm formation, we performed a time-course experiment in which cell lysis was blocked at various time points during development by the addition of polyanethole sulfonate (PAS), a chemical lysis inhibitor that does not affect growth [31], [32]. After 24 hours of biofilm development, the wells were washed and the amount of biofilm remaining was assessed by staining with crystal violet and quantifying the amount of stain that was retained [7]. Despite the presence of similar amounts of bacterial growth (data not shown), PAS treatment at the zero and two-hour time points resulted in a dramatic reduction in the amount of adherent biomass (Fig. 1) and correlates with lower eDNA levels measured from biofilms grown in the presence of PAS as previously reported [7]. In contrast, the addition of PAS as early as four hours post-inoculation had little effect on biofilm adherence. In parallel, biofilms were exposed to DNase I at each point during this time course experiment to gauge the contribution of eDNA to biofilm adherence. Unlike PAS treatment, the addition of DNase I at all time-points diminished cell adherence (Fig. 1) without having a demonstrable effect on bacterial growth (data not shown). These results indicate that cell lysis during the initial stage of biofilm development releases a sufficient amount of genomic DNA to mediate adherence. Furthermore, removal of this eDNA at any point during biofilm development in this static model of biofilm growth results in decreased biofilm adherence.

### Effect of *lrgAB* on biofilm development

**Figure 2:**
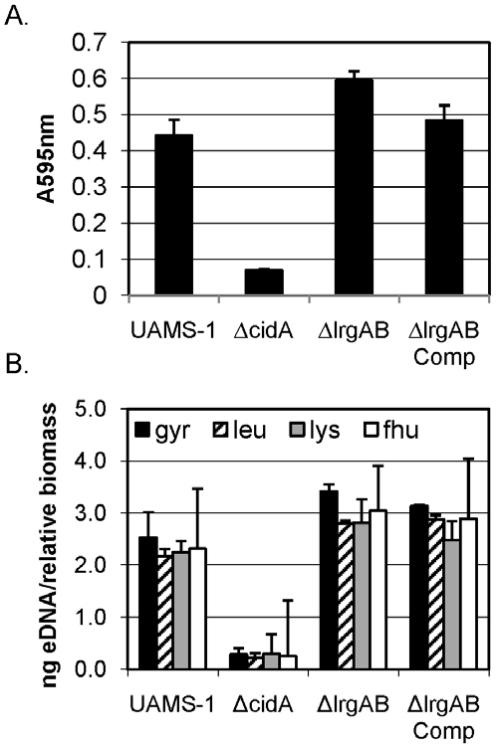
Static assays and eDNA quantification. *S. aureus* static biofilms were grown for 24 hours. (A) Washed biofilms were stained with crystal violet and quantified spectrophotometrically at an absorbance of 595 nm. The decrease in the *cidA* mutant (KB1050) biofilm adherence observed compared to the wild-type strain (UAMS-1) was statistically significant (p = 0.0004; ANOVA), as was the increased *lrgAB* mutant (KB1045) biofilm adherence (p = 0.021). There is no significant difference between the complementation strain (KB1046) (p = 0.39) and the UAMS-1 phenotype. (B) Extracellular DNA was isolated from the biofilm matrices of UAMS-1, KB1050, KB1045, and KB1046 and qRT-PCR of four chromosomal loci were amplified, *gyr* (black bars), *lue* (slashed bars), *lys* (grey bars), and *fhu* (white bars). The relative biomass was quantified at OD_600_, and the eDNA measurements were normalized to total biofilm biomass as described previously [7]. The relative concentration of eDNA decreases in the *cidA* mutant (p<0.0001) and increases in the in the *lrgAB* mutant (p = 0.020) compared to the wild-type. There is no significant difference between the complementation strain (KB1046) (p = 0.36) and the wild-type. Results are depicted as averages of three independent experiments and error bars represent the SEM.

Previous results from our laboratory [7] have also demonstrated the dependence of cell lysis during biofilm development on the *S. aureus cidA* gene encoding a known effector of murein hydrolase activity [18], [25], [33]. In the current study, we have extended our analysis of cell lysis to address the impact of the *cid* counterpart, *lrg*, on biofilm formation. After several unsuccessful attempts to demonstrate a reproducible phenotype of the *lrgAB* mutant in the static biofilm assay (unpublished data), we reasoned that the high level of NaCl (3.0% wt/vol) in the biofilm media may suppress any effect of *lrgAB* on the biofilm. We based this reasoning on data indicating that a high concentration of NaCl inhibits *lrgAB* expression [34] and because high NaCl levels are known to enhance autolysis [31]. Thus, static biofilm assays were repeated using medium lacking NaCl supplementation. Under these conditions, the *lrgAB* mutant (KB1045) biofilm displayed significantly enhanced adherence compared to the parental strain (Fig. 2). Furthermore, wild-type levels of biofilm adherence were restored in the complementation strain (KB1046) expressing *lrgAB* from a plasmid (Fig. 2A). As observed previously, the *cidA* mutant (KB1050) exhibited a reduced capacity for biofilm adherence compared to wild-type (Fig. 2A). As biofilm adherence was previously shown to be dependent on the amount of eDNA associated with the biofilm [7], we also measured the relative concentration of eDNA in each biofilm using quantitative real-time PCR (qRT-PCR). As observed previously [7], the *cidA* mutant biofilm contained significantly reduced levels of eDNA associated with the biofilm (Fig 2B). Conversely, the *lrgAB* mutant biofilm demonstrated significantly increased levels of eDNA compared to wild-type (Fig. 2). Importantly, the adherence and eDNA associated with the biofilm produced by the *lrgAB* complement were not significantly different compared to the wild-type strain. Overall, the results of these experiments suggest that the *lrgAB* operon has an inhibitory role in cell lysis and adherence during biofilm formation.

### Analysis of biofilm maturation

**Figure 3:**
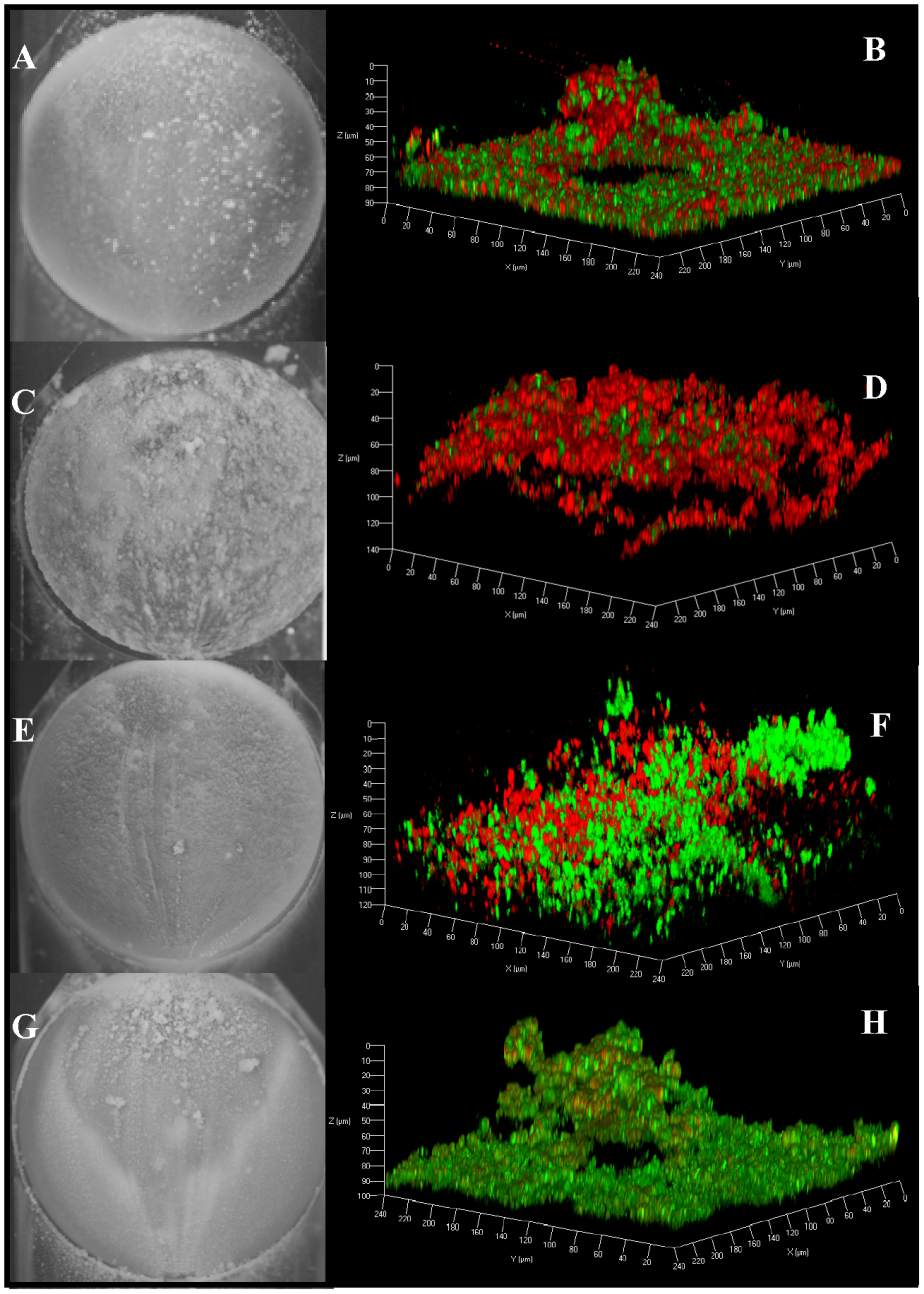
Flow cell biofilm assays. *S. aureus* 3-day biofilms were grown and representative images were taken using a macroscopic camera (panels A, C, E, and G) or CLSM (panels B, D, F, and H). The samples were stained with Syto-9 (green) and propidium iodide (PI; red) to indicate live and dead cell populations, respectively. The UAMS-1 (A and B), *cidA* mutant (C and D), *lrgAB* mutant (E and F), and *lrgAB* complemented (G and H) biofilms were grown for three days in the flow-cell model prior to imaging. The images shown are representative of four independent experiments.

Since static assays only measure the early events during biofilm development and not biofilm maturation, we tested the effects of the *cid* and *lrg* mutations during later stages of biofilm development by performing flow-cell experiments in which the biofilms were allowed to develop over a period of three days. As shown in Figure 3 (A & B), wild-type *S. aureus* (UAMS-1) produced biofilm with distinct structures randomly formed throughout the surface of the flow-cell chamber, reminiscent of the three-dimensional tower structures formed by other well-characterized biofilm producers [9], [11], [12], [17], [35]. Staining of the UAMS-1 biofilm with the LIVE/DEAD BacLight viability stain and visualization by CLSM revealed that the tower structures arose from and were surrounded by a flat mat or “lawn” of densely packed cells that was approximately 10 µm thick. The variation in the sizes of the structures formed is evident in macroscopic images taken after three days of biofilm growth (Fig. 3A). Although there is a clear indication of live (green) and dead (red) cells homogeneously scattered throughout the lawn, an increased concentration of dead cells localized within the towers is apparent (Fig 3B), similar to observations of *P. aeruginosa* biofilm [11]. The tower structures were not observed in static assays and were only visible in the flow-cell assays macroscopically after the second day of incubation (unpublished results). Furthermore, the flow-cell biofilm structures form abundantly using dilute TSBg media and an initial inoculum of approximately 5×10^5^ CFU. This is in contrast to flatter, less structured biofilms grown using higher inoculums to initiate biofilm growth, conditions similar to those used in the static biofilm assay. These observations are consistent with the observation that limiting nutrients allows *P. aeruginosa* biofilm to form better secondary structure [9].

In contrast to the parental UAMS-1 strain, the *cidA* (KB1050) and *lrgAB* (KB1045) mutants formed biofilms after three days growth that lacked obvious tower structures (Fig. 3; panels C-F). CLSM data demonstrated that the *cidA* and *lrgAB* mutants produced biofilms under these conditions that were homogeneous in nature, lacking the densely-packed lawn and tower structures that are characteristic of the wild-type biofilm. As shown in figure 3 (panels G & H), complementation of the *lrgAB* mutation partially restored the wild-type biofilm phenotype as indicated by the presence of tower structures and the characteristic dense, flat basal biofilm. However, as was observed with the static assay, complementation was incomplete as the tower structures were not as well defined and fewer dead cells were present. The visible abundance of the lawn in the UAMS-1 biofilm compared to the mutants agreed favorably with the roughness coefficients calculated using COMSTAT analysis of the CLSM images, which were 0.238±0.056 for the wild-type biofilm and 0.790±0.121 and 0.941±0.119 for the *cidA* and *lrgAB* mutants, respectively. The complemented strain generated a roughness coefficient that was intermediate between the wild-type and mutant strains (0.563±0.071) consistent with the observed architecture visualized in figure 3G and 3H. The average maximum thickness (determined from multiple images) of the biofilms produced by the *cidA* and *lrgAB* mutants (143.66±2.40 and 112.75±10.14 µm, respectively) were higher compared to the parental and *lrgAB* complemented strains (94.50±6.69 and 110.80±3.54 µm, respectively). In addition, the COMSTAT data indicated that the wild-type biofilm contained greater biomass (15.35±1.43 µm^3^/µm^2^) compared to the *cidA* and *lrgAB* mutant biofilms (8.71±1.56 µm^3^/µm^2^ and 4.80±0.56 µm^3^/µm^2^, respectively). As above, the reduced biomass generated by the *lrgAB* mutant was partially complemented (8.69±0.813 µm^3^/µm^2^) by the presence of the *lrgAB* expression from a plasmid. Finally, the CLSM images also revealed a pattern of LIVE/DEAD staining that was distinct in the mutant strains. Notably, the dead cells in the *cidA* mutant biofilm accumulated to high numbers (Fig. 3D), consistent with the observation that this mutant exhibits normal cell death in stationary phase planktonic cultures but does not lyse [36]. In contrast, the *lrgAB* mutant appeared to contain more live cells compared to the wild-type strain (Fig. 3; compare panels B & F). Combined, these observations provide further support for the importance of the *cid* and *lrg* operons during biofilm formation.

**Figure 4:**
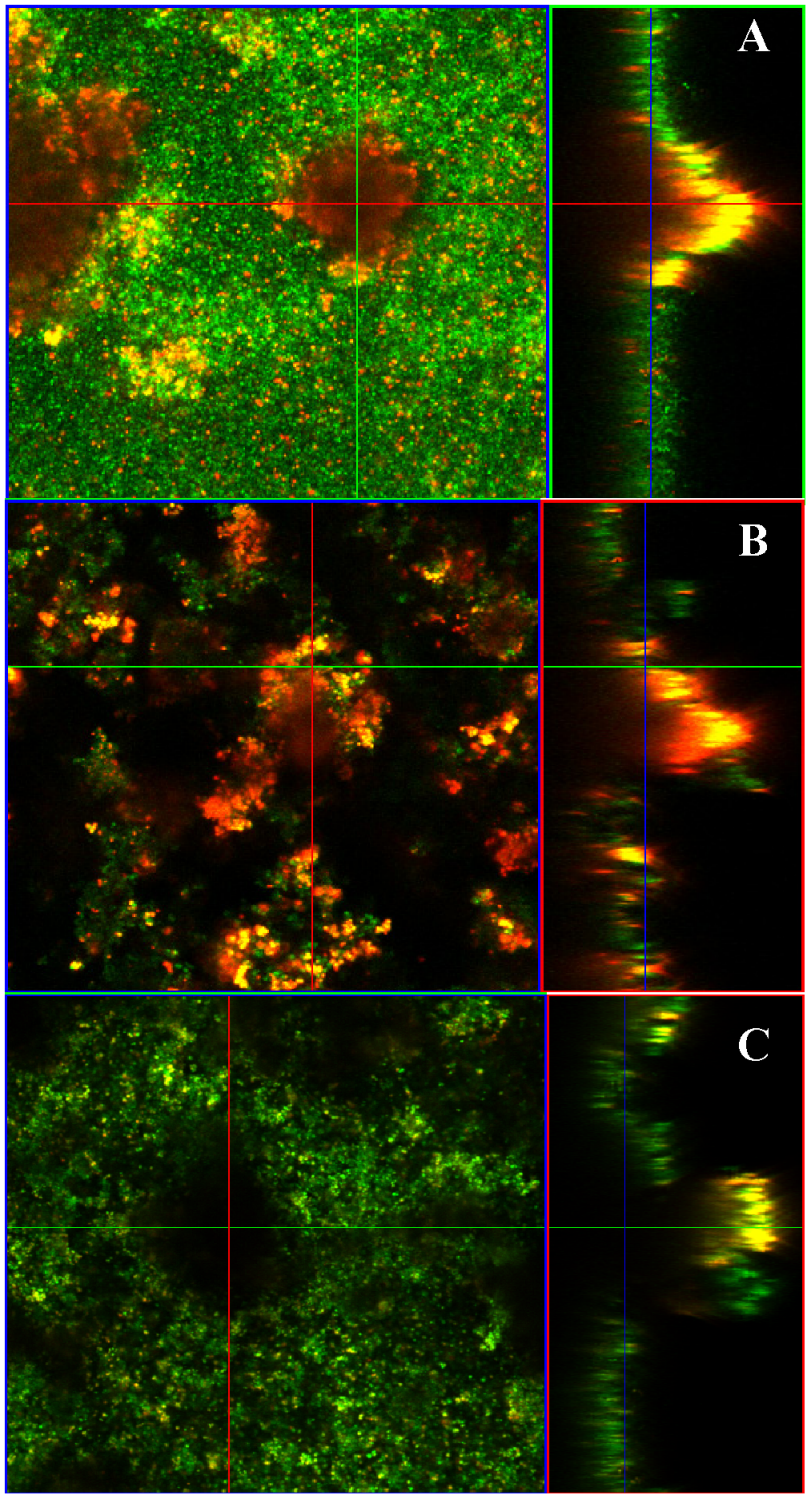
Visualisation of eDNA. *S. aureus* flow-cell biofilms were stained with Syto-9 and Toto-3 to indicate the location of live cells (green) and dead cells (punctuate red) and extracellular DNA (diffuse red). A yellow appearance is observed where live cells and eDNA are both present. The UAMS-1 (A), *cidA* mutant (B), and *lrgAB* mutant (C) biofilms were grown for three days prior to imaging and representative CLSM images are shown. The images shown are representative of three independent experiments.

Since the heterogeneous nature of the flow-cell biofilms made QRT-PCR quantification of eDNA difficult, we chose to visualize the eDNA produced within the biofilm using the nucleic acid-specific fluorophore, Toto-3. The advantage of this stain is that it has greater fluorescence enhancement compared to PI and Syto-9 when bound to nucleic acid, thereby facilitating visualization of eDNA by CLSM [37]. As illustrated in Figure 4 (panel A), Toto-3 staining was most prominent in the wild-type biofilm tower structures and was diffuse in nature suggesting that it is detecting eDNA. In contrast, the *lrgAB* mutant biofilm stained more intensely with Toto-3 throughout the biofilm consistent with the increased levels of eDNA detected in the static assays (Fig. 2). As observed in the previous experiment (Fig. 3), the dense 10 µm basal layer of biofilm and well-defined structures were also absent in the *lrgAB* mutant. Importantly, complementation of the *lrgAB* mutant resulted in Toto-3 staining that was more similar to the parental strain, although the diffuse staining associated with the towers was not observed (Fig. 4). Combined, these studies support the model in which the *cid* and *lrg* operons encode effectors and inhibitors of cell lysis, respectively, and that they are important for the control of DNA release during biofilm development [20], [38].

### Susceptibility of mature staphylococcal biofilm to DNase I

**Figure 5:**
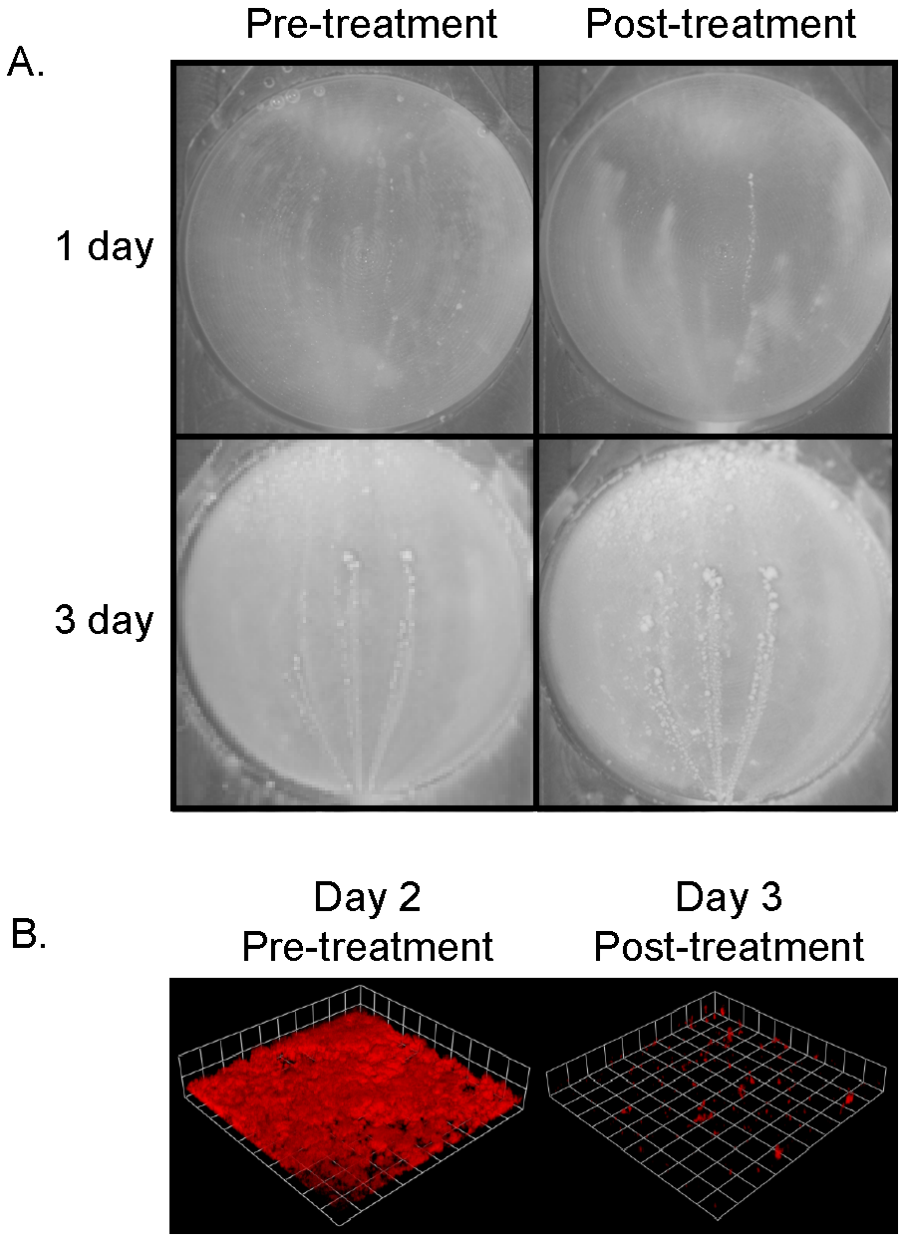
Mature biofilm sensitivity to DNase I. (A) *S. aureus* UAMS-1 biofilms were grown in the flow-cell model with low inoculum for one or three days. The images of untreated biofilms where taken immediately before DNase I exposure and the images of the same biofilms treated with DNase I were taken five hours later. (B) *S. aureus* UAMS-1 biofilms harboring a constitutively-expressed gene (*rfp*) encoding red fluorescence protein were grown in the same flow-cell model using a higher inoculum for two days. The biofilm was then treated with DNase I for a total of 24 hours after which CLSM z-stacks were taken. Renderings of the z-stack CLSM data were performed using the Volocity software.

In figure 1, we demonstrated that static biofilms are sensitive to DNase I treatment at any stage during the 24 hour growth period, indicating that eDNA is an important part of the biofilm matrix during the initial stages of biofilm development. To investigate the role of eDNA in more mature biofilm, *S. aureus* UAMS-1 was grown in the flow-cell biofilm model and treated with DNase I at both early (24 hr) and late (72 hr) time points. The biofilms were then allowed to grow for an additional six hours and visualized. As shown in figure 5, the biofilms exposed to DNase I were visibly disrupted compared to the biofilms imaged immediately prior to DNase I exposure. In contrast, biofilms treated with DNase I after 72 hr growth appeared to be largely resistant to DNase I when comparing pre- and post-DNase I treatment images (Fig. 5). Interestingly, wild-type biofilms initiated with higher inoculums (approximately 1×10^8^ cfu/ml versus 5×10^5^ cfu/ml), resulted in greater sensitivity to DNase I as demonstrated by the nearly complete removal of a three-day old biofilm as determined by CLSM (Fig. 5B). These results indicate that biofilm dispersal is impacted by the age of the biofilm as well as number of bacterial cells used to initiate biofilm growth, illustrating the fact that biofilms established using different conditions are distinct from each other.

### Staphylococcal nuclease effects biofilm maturation

**Figure 6:**
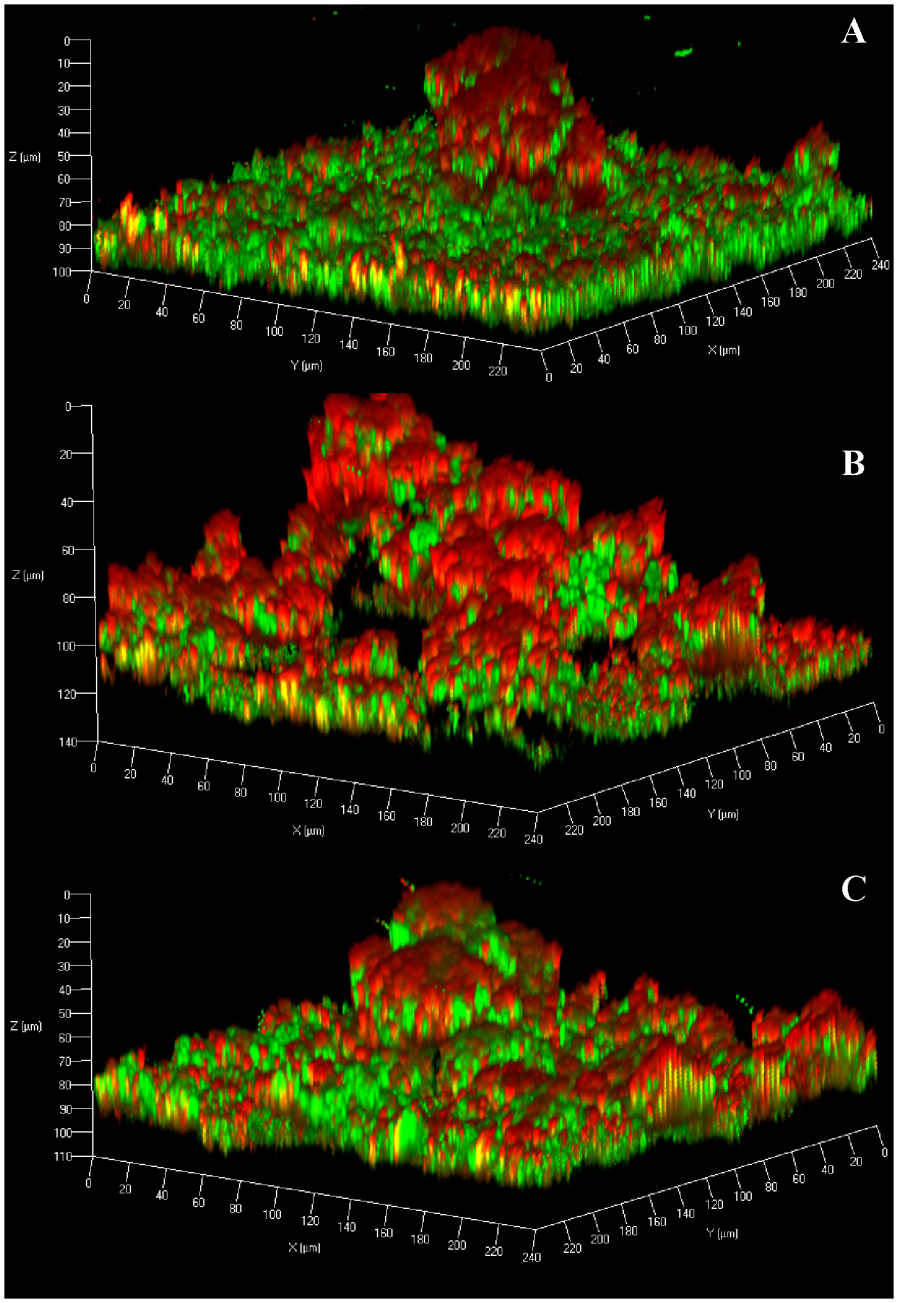
Staphylococcal thermonuclease affects biofilm structure. Representative CLSM z-stacks of UAMS-1 (A), *nuc* mutant (B), and *nuc* complemented (C) biofilms stained with Syto-9 (green) and Toto-3 (red). Syto-9 represents the live cells and Toto-3 represents dead cells and eDNA. The images shown are representative of three independent experiments.

Given the effect of exogenously added DNase I on *S. aureus* biofilm, we considered the possibility that staphylococcal thermonuclease has a previously unappreciated role in biofilm development. Indeed, the *nuc* gene encoding staphylococcal thermonuclease was found to be differentially regulated within a biofilm [39], [40] and was recently shown to diminish adherence of UAMS-1 biofilm under conditions where it is over-produced [41]. Therefore, we grew the recently characterized *nuc* mutant (UAMS-1471) in our flow-cell assay to determine the impact of staphylococcal thermonuclease on biofilm formation. Interestingly, the *nuc* mutant biofilm exhibited increased Toto-3 staining compared to the parental and complementation strains (Fig. 6). The increased fluorescence intensity can be attributed to increased eDNA levels in the matrix as dilution plating experiments revealed similar viable cell densities between these strains (data not shown). The *nuc* mutant also had a higher roughness coefficient (0.667±0.058) relative to the parental (0.238±0.056) and complementation strains (0.523±0.174) indicating that the altered level of eDNA had an significant effect on biofilm architecture. The effect of the *nuc* mutation was even more pronounced in biofilms initiated with higher inoculums, resulting in a doubling in thickness from approximately 10 µm to approximately 20 µm (Fig. S1). These results demonstrate that staphylococcal thermonuclease plays a significant role in biofilm development possibly by degrading the eDNA associated with the biofilm.

## Discussion

The *S. aureus cid* and *lrg* operons have been previously shown to have opposing effects on the control of murein activity and lysis in cells grown in planktonic culture. The *cidA* gene is an effector of these processes as indicated by the observation that a *cidA* mutant displays reduced murein hydrolase activity and stationary phase lysis [18], [25], [36]. In contrast, the *lrgAB* operon is associated with the inhibition of murein hydrolase activity [19]. Combined with the similarities of the *cidA* and *lrgA* gene products to bacteriophage-encoded holins, it has been proposed that these proteins function in a manner similar to holins and antiholins, respectively [18], [20], [21], [42]. The results of the current study support this model by demonstrating the opposing effects of the *cid* and *lrg* operons in the control of cell lysis during biofilm development and provide additional insight into factors modulating the release and metabolism of genomic DNA within a biofilm.

Building on the observation that an inhibitor of lysis, PAS, reduced genomic DNA release and biofilm adherence [7], we assessed the temporal requirements for lysis during the early stages of biofilm development. As shown in figure 1, addition of PAS within the first four hours of development resulted in the reduced capacity of the biofilm to adhere to the surface of microtiter wells. Interestingly, this effect of PAS was lost after the biofilm was greater than four-hours old, presumably due to the accumulation of sufficient levels of eDNA in the biofilm matrix to allow for adherence throughout the entire time-course of the experiment. Although more mature flow-cell biofilms were also sensitive to DNase I treatment, the effect diminished after three days of growth (Fig. 5), suggesting that the biofilm matrix either becomes less dependent on eDNA over time, or that the matrix becomes resistant to the effects of the DNase I treatment. Recently published results, however, demonstrate that protease treatment can also disrupt a mature biofilm [3], [41], indicating that a proteinaceous component also exists in the biofilm matrix that either protects the eDNA from degradation or independently maintains mature biofilm adherence.

In addition, this is the first study to examine the effect of *lrgAB* on biofilm development. Since the *lrg* operon is the inhibitory counterpart to *cid*, an *lrg* mutation would be expected to produce an opposing phenotype compared to that of a *cid* mutation. Indeed, the *lrgAB* mutant exhibited enhanced biofilm adherence and increased eDNA associated with the biofilm matrix relative to the parental and complemented strains (Figs. 2, 3, and 4). In the static assay, this phenotype was found to be partially dependent on the level of NaCl used in the assay media, likely due to the repressive effects that NaCl has on *lrgAB* expression (unpublished results) and/or the stimulatory effects it has on lysis [31]. Importantly, these data are consistent with the proposed role of *lrgA* gene product as an antiholin [18], [19], inhibiting the activity of the *cidA*-encoded holin and allowing increased activity of murein hydrolases.

Another finding of this study was that tower structures, which formed in more mature biofilms, were associated with what appeared to be large quantities of eDNA (Fig. 4). Our CLSM data revealed that the internal regions of the tower structures are devoid of intact cells and are comprised, in part, of eDNA (diffuse red staining) beneath a layer containing a combination of dead cells and eDNA (intense red staining) (Fig. 4A). Interestingly, the tower structures exhibited a lack of staining near the base suggesting that the eDNA in this region has either diffused away, or has been degraded, forming a microenvironment lacking bacteria or eDNA (Fig. 4A). These results are consistent with observations of *P. aeruginosa* and *S. epidermidis* biofilms, which also form hollow voids within tower structures [11], [43]. Furthermore, the preponderance of eDNA in towers has also been observed in *P. aeruginosa* biofilms [11] and mutations in the *cid* and *lrg* homologues of this organism also had opposing effects on cell lysis during biofilm development [44].

In previous studies, our laboratory has described regulatory signals and transcription factors that control *cid* and *lrg* expression during planktonic growth [7], [24], [25], [45]. During biofilm development, however, it has been hypothesized that the expression of the *cid* and *lrg* operons is differentially regulated within individual cells in the biofilm population and that this control dictates which cells are destined for death and lysis and, on the other hand, which cells remain viable [38]. For example, this model predicts that those cells expressing high levels of *cid* and low levels of *lrg* will ultimately die and lyse, while those expressing low levels of *cid* and high levels of *lrg* will remain viable. Importantly, the balance between these two opposing operons may be critical in determining the proportion of live and dead cells within a biofilm [20]. Furthermore, the “tipping point” may vary depending on the environmental conditions. Indeed, recent data suggests that staphylococcal biofilms are comprised of distinct anaerobic microenvironments [46] and that cell lysis is enhanced within the anaerobic portions of the biofilm (Philip Stewart; personal communication). Recent studies also indicate that *cidA* expression is induced under anaerobic conditions and that this leads to cell lysis in these regions of the biofilm (data not shown). Whether or not known regulators of *cid* and *lrg* expression, such as LytSR and CidR [22], [23], [45], [47], are involved in the control of their expression within a biofilm remains to be determined.

In general the environmental conditions under which biofilms are grown have a dramatic effect on biofilm development. After several attempts to optimize biofilm structure formation in *S. aureus*, we observed that a more dilute starting inoculum aided the generation of tertiary structure within the biofilm. The lower inoculum led to pronounced tertiary structure formation in biofilms produced by UAMS-1 (Figs. 3 and 4), as well as those produced by USA300 LAC (data not shown). It is hypothesized that growth of biofilms using a high inoculum may subvert tower formation due to the high density of cells on the substratum, resulting in thicker initial biofilms. In contrast, the propagation of biofilms using low inoculums may allow the biofilm to mature more gradually, allowing both temporal and spatial signals needed for secondary structure formation. The initial cell density on the substratum may also explain why “low inoculum” biofilms are more resistant to DNase I treatment at the three-day time point in comparison to “high inoculum” biofilms. For example, since the initial steps of biofilm adherence are lysis dependent (Fig. 1), increased numbers of cells used to initiate biofilm formation may lead to a proportionally increased number of cells undergoing cell lysis and, consequently, an enhanced dependence of biofilm adherence on eDNA and increased sensitivity to DNase I treatment. Alternatively, the greater concentration of cells on the substratum may provide an environment promoting cell lysis and DNA release due to the metabolic activity of thick biofilms [46]. Regardless of the mechanism, continued investigations should lead to important information related to the initial events needed for biofilm development.

Because of the sensitivity of the *S. aureus* biofilm to DNase I, we explored the possibility that staphylococcal thermonuclease may play a role in biofilm development. This is consistent with the recent finding that *nuc* makes a significant contribution to the biofilm deficient phenotype of a *sarA* mutant [41]. In the current study, biofilms produced by the *nuc* mutant (UAMS-1471) were thicker and contained increased levels of eDNA in the matrix compared to the parental and complemented strains (Fig. 6). Importantly, these phenotypes were observed in biofilms produced using both low (Fig. 6) and high initial inoculums (Fig. S1). Thus, these results suggest that the lysis-mediated release of genomic DNA may be balanced by the active degradation of eDNA by thermonuclease, leading to an optimized level of eDNA associated with the biofilm. Whether this activity is also important in the dispersal of cells from the biofilm, as has been well-documented in *S. aureus* [3], [48], remains to be determined.

Overall, the results of this study provide further insight into the regulatory mechanisms controlling cell death and lysis during biofilm development and are consistent with the previously proposed functions of the *cid* and *lrg* operons in the control of cell lysis. Furthermore, these studies support the role of genomic DNA in biofilm formation and suggest that its degradation may be an important aspect of biofilm maintenance that was not previously recognized. Continued studies of the Cid/Lrg system, including the analysis of the metabolic, temporal, and spatial factors that affect expression of this system should be important for gaining a full understanding of biofilm formation and the specific roles of these proteins in this process.

## Supporting Information

Figure S1 High inoculum nuclease mutant biofilms. Biofilms of UAMS-1, nuc mutant, and nuc complemented strains each harboring pAH9 conferring RFP fluorescence where initiated with a 1:100 inoculum. The three-day biofilms of each were imaged using CLSM and z-stacks were rendered using Volocity software.
(0.74 MB TIF)
