# Supplementary figures and images for: Modulation of eDNA Release and Degradation Affects Staphylococcus aureus Biofilm Maturation
Source: PLoS One. 2009 Jun 9;4(6):e5822. doi: 10.1371/journal.pone.0005822 (PMC2688759; doi:10.1371/journal.pone.0005822)

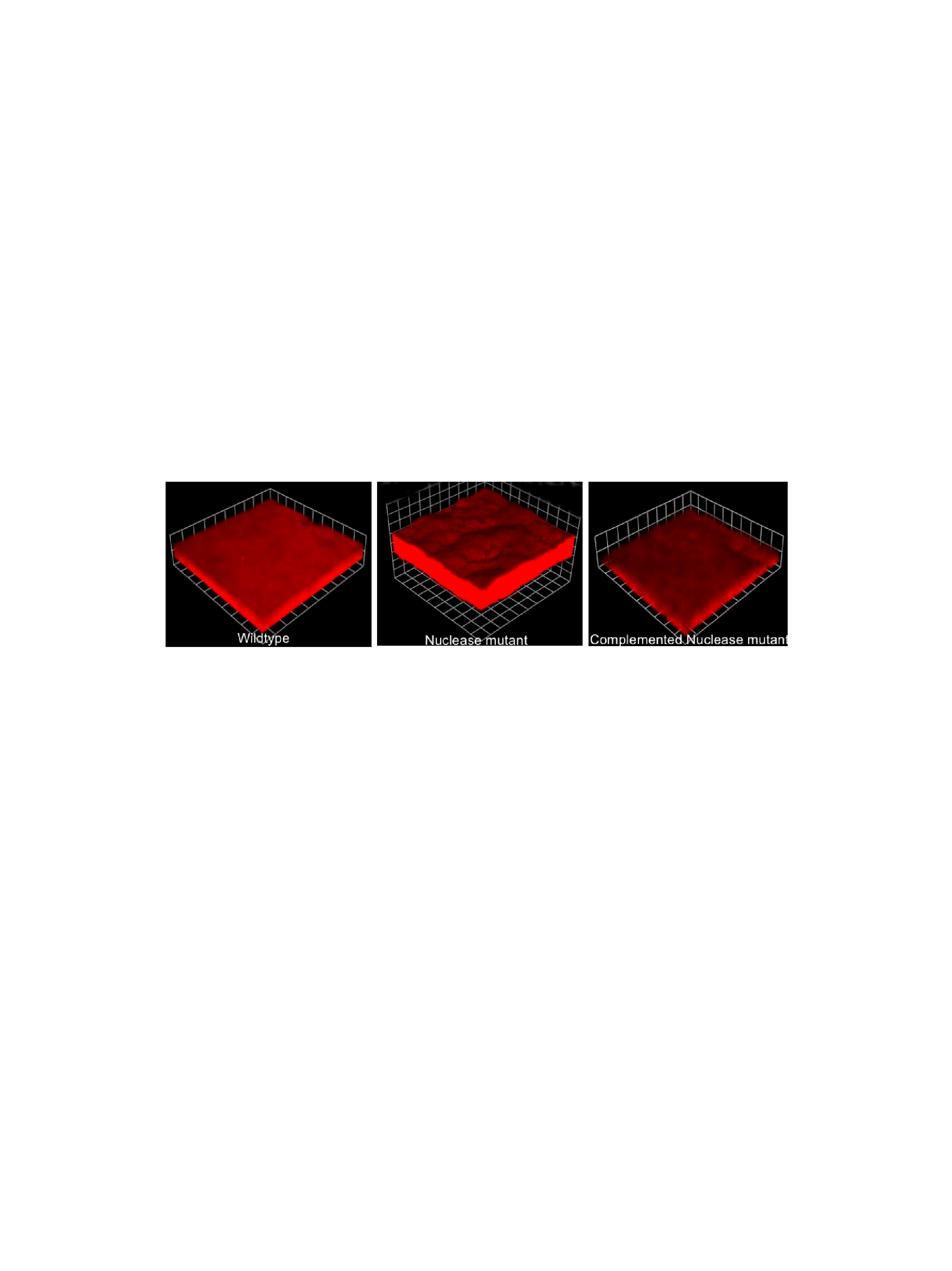

Supplement: Figure S1 — High inoculum nuclease mutant biofilms. Biofilms of UAMS-1, nuc mutant, and nuc complemented strains each harboring pAH9 conferring RFP fluorescence where initiated with a 1:100 inoculum. The three-day biofilms of each were imaged using CLSM and z-stacks were rendered using Volocity software. (0.74 MB TIF) [file pone.0005822.s001.tif]
